# The expressions and m^6^A methylation modification patterns of mRNAs and lncRNAs in major depressive disorder

**DOI:** 10.3389/fgene.2026.1850052

**Published:** 2026-09-11

**Authors:** Liang Liu, Zhaonan Yang, Kunru Tan, Shuhao Xu, Jia Xu, Jinbao Niu, Xiuxian Yang

**Affiliations:** 1 Department of Medical Psychology, Center for Psychological Science and Health Management, Harbin Medical University, Harbin, China; 2 Department of Psychology and Sleep Medicine, The First Psychiartric Hospital of Harbin, Harbin, China

**Keywords:** epitranscriptomic, lncRNA, m6A methylation, major depressive disorder (MDD), mRNA

## Abstract

**Objective:**

To systematically characterize the epitranscriptomic landscape of N^6^-methyladenosine (m^6^A) methylation in major depressive disorder (MDD) and elucidate its functional consequences on mRNA and lncRNA regulation.

**Methods:**

We performed comprehensive m^6^A methylome profiling using microarray analysis of peripheral blood samples from first-episode, treatment-naïve MDD patients (n = 4) and matched healthy controls (n = 4). Differentially methylated RNAs were identified through stringent criteria (fold-change ≥1.5, probability value (p) < 0.05), followed by integrated bioinformatics analyses including Gene Ontology (GO) and Kyoto Encyclopedia of Genes and Genomes (KEGG) pathway analysis. Validation of selected RNAs (*GRM4, CAMKK2, TTC28-AS1*, and *PAX8-AS1*) was performed using MeRIP-qPCR in 15 MDD patients and 15 controls.

**Results:**

Significant differences in m^6^A methylation of mRNAs and lncRNAs were observed between MDD patients and healthy controls. 513 mRNAs (180 hypermethylated, 333 hypomethylated) and 119 lncRNAs (58 hypermethylated, 61 hypomethylated) showed differential m^6^A modifications. GO and KEGG analyses revealed that hypermethylated mRNAs were mainly involved in neuroactive ligand-receptor interactions, while hypomethylated mRNAs were associated with the AMPK signaling pathway. Among the 350 differentially expressed mRNAs, 171 were upregulated and 179 downregulated, enriched in cAMP and TNF signaling pathways, respectively. Among 110 differentially expressed lncRNAs, 71 were upregulated and 39 downregulated. Validation confirmed significant m^6^A methylation differences in *GRM4* (p < 0.001), *CAMKK2* (p = 0.012), and *PAX8-AS1* (p = 0.006), but not significantly different in *TTC28-AS1*.

**Conclusion:**

Our findings suggest that peripheral m^6^A methylation signatures are associated with MDD pathophysiology, with *GRM4* and *PAX8-AS1* representing promising candidate biomarkers, while the AMPK and neuroactive ligand-receptor pathways provide hypothesis-generating avenues for future mechanistic investigation.

## 1 Introduction

Major depressive disorder (MDD) is one of the most prevalent psychiatric diseases worldwide, characterized by persistent low mood and anhedonia (Shi et al., 2011). According to the World Health Organization, over 350 million individuals currently suffer from depressive disorders, with incidence rates increasing by nearly 30% since the onset of the COVID-19 pandemic (World Health Organization, 2025). MDD poses a critical public health challenge due to its high prevalence, elevated recurrence rates, chronic course, frequent comorbidity with physical illnesses, and substantial disease burden (Kessler et al., 2003).

The onset and progression of depression are influenced by a combination of genetic and environmental factors (Torres-Berrío et al., 2019). Epigenetics, defined as the process by which nucleic acids undergo chemical modifications in response to stress and environmental stimuli (Meaney, 2010), focuses on heritable changes in gene expression that occur without alterations to the underlying DNA sequence, ultimately leading to phenotypic changes. Key epigenetic mechanisms include DNA methylation, RNA methylation, histone acetylation and methylation, and microRNA regulation. While DNA methylation studies have provided important insights into MDD epigenetics, recent large-scale meta-analyses have revealed that no significant global DNA methylation differences exist between MDD cases and controls, instead identifying rare epivariations and locus-specific changes such as hypomethylation near the *SHF* gene (Baldrighi et al., 2024). These findings suggest that DNA methylation alterations in MDD may be relatively focal rather than genome-wide. In contrast, the role of RNA modifications, particularly m^6^Amethylation, in MDD pathophysiology remains less explored but is gaining increasing attention (Mitsuhashi et al., 2024).

N^6^-methyladenosine (m^6^A) methylation is the most prevalent internal modification in mammalian messenger RNA (mRNA), occurring at approximately 0.1%–0.6% of all adenosine residues (Cappannini et al., 2024). This modification predominantly localizes within the DRACH consensus sequence (D = A/G/U; R = A/G; H = A/C/U), with notable enrichment near stop codons and 3′untranslated regions (3′UTRs). m^6^A modification is dynamically regulated by writers, including methyltransferase-like 3 (*METTL3*), methyltransferase-like 14 (*METTL14*), and WT1-associated protein (WTAP), and erasers, including fat mass and obesity-associated protein (*FTO*) and *ALKB* homolog 5 (*ALKBH5*). Functionally, m^6^A exerts pleiotropic effects on RNA metabolism through selective recognition by *YTH* domain-containing reader proteins, which modulate mRNA stability and degradation, translation efficiency, alternative splicing patterns, and nuclear–cytoplasmic shuttling (Fu et al., 2014).

Accumulating evidence indicates that m^6^A methylation exerts broad effects on the nervous system, influencing neural stem cell self-renewal, learning and memory, brain development, and synaptic growth. Notably, m^6^A modification has been closely linked to brain activity and psychiatric disorders (Jiang et al., 2022). A previous study investigated the distribution of m^6^A in the brains of adult mice subjected to stress-induced depression and found that stress altered global m^6^A/mRNA levels (Engel et al., 2018). In parallel, global m^6^A methylation in human and mouse whole blood was found to decrease following acute stress. Furthermore, numerous studies have reported altered expression of RNA methyltransferases (*METTL3, METTL14,* and *WTAP*) and demethylases (*FTO* and *ALKBH5*) in MDD patients and mouse models of depression (Liu et al., 2021). Astrocytic *ALKBH5* has been implicated in stress responses contributing to depressive-like behaviors in mice, and *FTO* has been shown to regulate RNA methylation associated with depression-like phenotypes. These findings collectively suggest that dysregulation of m^6^A modification may underlie the pathophysiology of depression, as evidenced by altered m^6^A/mRNA levels in the hippocampus, prefrontal cortex, and peripheral blood of depressed animal models and MDD patients. However, the precise role of m^6^A modification, particularly its impact on both mRNAs and long non-coding RNAs (lncRNAs), in the pathogenesis of MDD remains poorly understood.

Long non-coding RNAs (lncRNAs) have emerged as critical regulators of gene expression and are increasingly implicated in the pathogenesis of various complex human diseases, including cardiovascular disorders, autoimmune conditions, neurodegenerative diseases, and malignancies (Kumar et al., 2024). Within the central nervous system, lncRNAs modulate key biological processes including neuronal differentiation, synaptic plasticity, stress responses, and neuroinflammation, which are intimately linked to the pathophysiology of major depressive disorder (MDD) (Roy et al., 2025). Notably, aberrant lncRNA expression has been detected in the peripheral blood of MDD patients, with candidates such as *HYMAI* and *RMRP* showing promise as non-invasive diagnostic biomarkers or correlating with clinical symptom severity (Seki et al., 2019; Bu et al., 2025). Beyond their biomarker potential, the dynamic and reversible nature of m^6^A methylation raises the possibility that these epitranscriptomic marks could be modulated through therapeutic interventions. By linking m^6^A methylation of specific lncRNAs to well-characterized signaling cascades, particularly neuroactive ligand-receptor interactions and the AMPK pathway, this approach may provide a molecular framework for the development of targeted strategies aimed at correcting epitranscriptomic dysregulation in MDD. If validated in larger clinical cohorts, these insights may ultimately inform personalized diagnostic and therapeutic approaches for MDD patients.

In this study, we aimed to investigate the relationship between m^6^A methylation-related genes and depression, and elucidate the mechanisms by which m^6^A modification of mRNAs and lncRNAs contributes to MDD. To this end, we first performed microarray-based m^6^A methylome profiling on immunoprecipitated methylated RNA from peripheral blood samples of MDD patients and healthy controls. Subsequently, we employed comprehensive bioinformatics analyses to explore the potential pathogenic pathways associated with m^6^A methylation in MDD.

## 2 Materials and methods

### 2.1 Participants

Fifteen patients were recruited from the First Psychiatric Hospital of Harbin between September 2022 and March 2023. All patients were diagnosed with major depressive disorder by two psychiatrists based on structured interviews using the Diagnostic and Statistical Manual of Mental Disorders, Fifth Edition (DSM-5). Depression severity was assessed using the Chinese version of the 17-item Hamilton Depression Rating Scale (HAMD-17) (Zimmerman et al., 2013), and patients with a total score of ≥17 were included. All patients were experiencing their first depressive episode and were treatment-naïve, with no prior history of antidepressant medication.

A total of 15 healthy control participants were recruited from the Physical Examination Center of the First Affiliated Hospital of Harbin Medical University. A psychiatrist conducted structured interviews to rule out MDD in the control group. Controls were frequency-matched to MDD patients by age and sex.

All participants were of Han Chinese descent. Exclusion criteria for both groups included other psychiatric disorders, intellectual disability, dementia, comorbid physical illnesses, family history of genetic disorders, history of alcohol or drug abuse, and major neurological diseases.

Family history information was obtained through a structured interview conducted by two psychiatrists at enrollment. Participants were asked about the presence of genetic disorders, alcohol or drug abuse, or major neurological diseases among their first-degree relatives (parents, siblings, and offspring). A family history was considered positive if any first-degree relative had been diagnosed with any of these conditions. Participants with a positive family history for these disorders and with known inflammatory diseases were excluded from both the MDD and control groups.

The study protocol was approved by the Ethics Committee of Harbin Medical University and was conducted in accordance with the ethical standards of the Declaration of Helsinki. Written informed consent was obtained from all participants before enrollment.

No significant differences were observed between MDD and control groups in age, sex, educational level, family history, or body mass index (BMI) (all p > 0.05). Additional clinical information, including smoking status and alcohol consumption, was obtained through structured interviews at enrollment, and no significant differences were found between the two groups for these variables (both p > 0.05). The duration of depressive symptoms was also recorded for MDD patients. Demographic and clinical characteristics of the participants are summarized in Table 1.

**TABLE 1:** Participants characteristics.

Characters	MDD group	Control group	P Value
Gender (male/female)	6/9	6/9	1.000
Age	41.00 ± 1.58	42.00 ± 2.24	0.147
Education (year)	14.40 ± 1.14	13.40 ± 2.07	0.112
Family history (y/n)	0/15	0/15	1.000
BMI	22.37 ± 0.67	22.72 ± 0.58	0.132
HAMD-17	32.60 ± 4.16	3.31 ± 1.26	0.000
Smoking status (y/n)	2/13	3/12	1.000
Alcohol consumption (y/n)	0/15	0/15	1.000
Duration of depressive symptoms (weeks)	6.81 ± 1.26	​	​

n = 15 per group (MDD, and healthy controls), Student’s t-test was used for continuous variables such as age, education years, BMI, HAMD, chi-square test for categorical variables including gender, family history, smoking status, and alcohol consumption. MDD, major depressive disorder; BMI, body mass index; HAMD-17, 17-item Hamilton Depression Rating Scale.

### 2.2 Extraction of total RNA

Peripheral venous blood samples (5 mL) were collected from participants under fasting conditions between 6:00 and 10:00 a.m. Plasma was separated by centrifugation at 1,000 × g for 15 min at 4 °C. The supernatant was transferred to RNase/DNase-free tubes and stored at −80 °C until further processing.

Total RNA was extracted from plasma using the miRNeasy Serum/Plasma Kit (Qiagen, United States) according to the manufacturer’s instructions. RNA concentration and purity were assessed using a NanoDrop ND-1000 spectrophotometer (Thermo Fisher Scientific, United States).

### 2.3 m^6^A immunoprecipitation

Total RNA (3–5 µg) was combined with an m^6^A spike-in control mixture and added to 300 µL of 1× immunoprecipitation (IP) buffer (50 mM Tris-HCl, pH 7.4, 150 mM NaCl, 0.1% NP-40, 40 U/µL RNase inhibitor), along with 2 µg of anti-m^6^A rabbit polyclonal antibody (Synaptic Systems). The reaction mixture was incubated with head-over-tail rotation at 4 °C for 2 h.

For isolation of antibody-bound RNA, 20 µL of Dynabeads™ M-280 Sheep Anti-Rabbit IgG suspension per sample was blocked with freshly prepared 0.5% bovine serum albumin (BSA) at 4 °C for 2 h. The beads were then washed three times with 300 µL of 1× IP buffer and resuspended in the total RNA-antibody mixture. RNA binding to the m^6^A-antibody-conjugated beads was performed with head-over-tail rotation at 4 °C for an additional 2 h.

Following binding, the beads were washed three times with 500 µL of 1× IP buffer and twice with 500 µL of wash buffer (50 mM Tris-HCl, pH 7.4, 50 mM NaCl, 0.1% NP-40, 40 U/µL RNase inhibitor). Enriched RNA was eluted with 200 µL of elution buffer (10 mM Tris-HCl, pH 7.4, 1 mM EDTA, 0.05% SDS, 40 U Proteinase K, and 1 µL of RNase inhibitor) at 50 °C for 1 h. Finally, RNA was extracted using acid phenol-chloroform and precipitated with ethanol.

### 2.4 Microarray labeling and hybridization

Following m^6^A immunoprecipitation, the RNA sample was separated into two fractions: the immunoprecipitated (IP) fraction (containing m^6^A-methylated RNA fragments bound to the anti-m^6^A antibody) and the supernatant (Sup) fraction (containing the unbound RNA fragments that lack m^6^A methylation). The “IP” RNAs and “Sup” RNAs were each spiked with equal amounts of calibration control RNA, then amplified and labeled with fluorescent dyes using the Arraystar Super RNA Labeling Kit (Sup: Cy3; IP: Cy5). The synthesized complementary RNAs (cRNAs) were purified using the RNeasy Mini Kit, and their concentrations and specific activities (pmol dye/μg cRNA) were measured with a NanoDrop ND-1000.

For hybridization, 2.5 μg of both Cy3-and Cy5-labeled cRNAs were mixed together. The cRNA mixture was fragmented by adding 5 μL of 10× Blocking Agent and 1 μL of 25× Fragmentation Buffer. This mixture was then heated at 60 °C for 30 min and combined with 25 μL of 2 × Hybridization Buffer. A total of 50 μL of the hybridization solution was dispensed onto the gasket slide and assembled with the m^6^A-mRNA&lncRNA Epitranscriptomic Microarray slide. The slides were incubated at 65 °C for 17 h in an Agilent Hybridization Oven. Finally, the hybridized arrays were washed, fixed, and scanned using an Agilent Scanner G2505C.

### 2.5 Microarray data processing

Array images were processed using Agilent Feature Extraction software (version 11.0.1.1). Raw signal intensities of IP (Cy5-labeled) and Sup (Cy3-labeled) samples were normalized to the average log_2_-transformed intensities of spike-in RNAs.

Calculation of expression and methylation levels: Because the Sup fraction retains the representation of all RNA transcripts independent of their methylation status, its signal intensity is proportional to total transcript abundance. Therefore, mRNA expression levels were calculated directly from the normalized Sup signal intensities. In contrast, m^6^A methylation levels for each transcript were determined by calculating the percentage of modification using the normalized intensities of both fractions: % m^6^A = IP/(IP + Sup).

### 2.6 Bioinformatics analysis

Differentially m^6^A-methylated and differentially expressed (DME) mRNAs were subjected to Gene Ontology (GO) and Kyoto Encyclopedia of Genes and Genomes (KEGG) pathway enrichment analyses using the cluster Profiler R package (Ashburner et al., 2000; Kanehisa and Goto, 2000). To predict the potential functions of differentially expressed long non-coding RNAs (lncRNAs), cis-regulated target genes were identified as mRNAs located within 100 kb upstream or downstream of the lncRNAs. Enrichment significance was assessed using Fisher’s exact test. Results were transformed into enrichment scores by calculating–log_10_(P). A false discovery rate (FDR)-adjusted p-value <0.05 and a fold change (FC) ≥1.5 were applied as thresholds for statistical significance.

### 2.7 Statistical analysis

Microarray differential analysis. To identify differentially m^6^A-methylated and differentially expressed transcripts between MDD and control groups, we employed the Limma package (version 3.50.0) in R with the empirical Bayes moderation method. The Limma model fits a linear model for each probe and borrows information across all probes to obtain more stable standard errors, which is particularly suitable for studies with small sample sizes (Ritchie et al., 2015; Smyth, 2004).

Fold changes and p-values between the MDD and control groups were computed for both m^6^A methylation levels (using IP/Sup ratio) and mRNA expression levels (using Sup signal). Given the exploratory nature of this pilot study with a small sample size (n = 4 per group) in the discovery microarray phase, we adopted a sensitivity-oriented screening strategy that prioritized the identification of potential biological signals over strict control of false positives. Transcripts with a fold change ≥1.5 and nominal p < 0.05 were selected for further bioinformatics analysis. While we acknowledge that this approach increases the risk of false-positive findings, it is a commonly used strategy in pilot epitranscriptomic studies to avoid excluding biologically relevant candidates that may not survive stringent multiple-testing correction due to limited statistical power. FDR was nevertheless calculated using the Benjamini–Hochberg method and is reported for transparency, but was not used as a primary inclusion criterion. Candidate transcripts identified through this permissive screening strategy were subsequently validated by MeRIP-qPCR in an independent cohort (n = 15 per group). Therefore, the results from the discovery phase should be interpreted as hypothesis-generating rather than confirmatory, with the primary goal being the identification of candidate m^6^A-methylated transcripts for validation rather than definitive inference.

Experimental validation and group comparisons. All MeRIP-qPCR experiments were performed in at least three independent replicates. Data are presented as mean ± standard deviation (SD). Normality of the data distribution was assessed using the Shapiro-Wilk test (p > 0.05 indicating normality). Statistical significance between the MDD and control groups was evaluated using Student’s t-test (for normally distributed data) or Mann-Whitney U test (for non-normally distributed data), with p < 0.05 considered statistically significant. All reported p-values are from two-tailed tests. Differences in demographic variables (age, BMI, HAMD, educational level) between groups were assessed using Student’s t-test, while sex and family history distribution were compared using the chi-square test.

## 3 Results

### 3.1 Transcriptome profile of m^6^A methylation in MDD (identification of differentially expressed or methylated genes)

**FIGURE 1:**
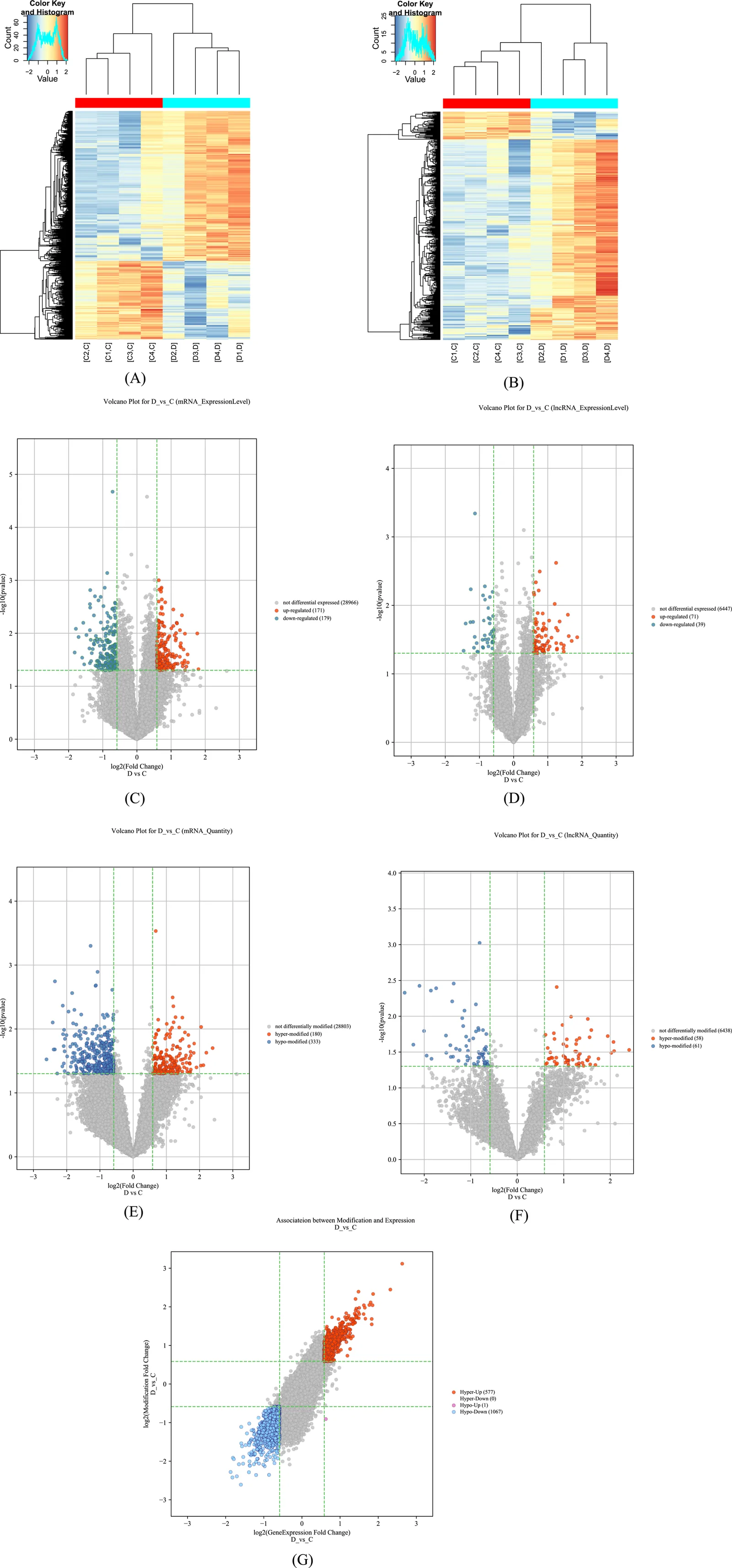
Different expression profiles of lncRNAs and mRNAs between MDD and control group. **(A)** Differentially methylated mRNAs. **(B)** Differentially methylated lncRNAs. Note: In Figure A and B, rows represent individual transcripts, and columns represent individual samples (n = 4 per group). The color scale (shown at the top) represents the normalized methylation level, ranging from blue (low) to red (high). The histogram above the color key displays the distribution of data values. Sample IDs: D1, D2, D3 and D4 correspond to MDD patients (n = 4); C1, C2, C3 and C4 correspond to healthy controls (n = 4). The dendrograms on the left and top indicate hierarchical clustering of transcripts and samples, respectively, performed using Euclidean distance and the complete linkage method. **(C)** Volcano plot of differential expression of mRNAs. The red dots indicate mRNAs with upregulated expression, and the blue dots represent mRNAs with downregulated expression. **(D)** Volcano plot of differential expression of lncRNAs.The red dots indicate lncRNAs with upregulated expression, and the blue dots represent lncRNAs with downregulated expression. **(E)** Volcano plot of differential mRNA methylation. The red dots indicate mRNAs with hyper-methytlation, and the blue dots represent mRNAs with hypo-methytlation. **(F)** Volcano plot of differential lncRNA methylation. The red dots indicate lncRNAs with hyper-methytlation, and the blue dots represent lncRNAs with hypo-methytlation. Note: n = 4 per group (MDD and healthy controls). Volcano plots display statistical significance (typically–log_10_ of p-value) against log_2_-transformed fold change (log_2_FC) on the Y and X-axes, respectively. In all volcano plots (Figures 1C–1F), upregulated/downregulated (for expression) or hypermethylated/hypomethylated (for methylation) transcripts were defined by fold change ≥1.5 and p < 0.05. **(G)** Association between RNA m^6^A modification levels and mRNA expression. The red dots indicate mRNAs with hyper-up expression; the blue dots represent mRNAs with hypo-down expression and purple dot demonstrates mRNAs with hypo-up expression. Note: n = 4 per group (MDD and healthy controls). Quadrant plot illustrating the relationship between m^6^A methylation changes (X-axis) and mRNA expression changes (Y-axis). Permissive filtering (fold change ≥1.5 for both methylation and expression, without p-value requirement) was applied to maximize sensitivity for detecting concordant directional patterns. Hyper/Up: hypermethylated and upregulated (577); Hypo/Down: hypomethylated and downregulated (1,067).

To investigate the role of m^6^A methylation in major depressive disorder (MDD), we performed epitranscriptomic microarray analysis to profile m^6^A methylation of mRNAs and lncRNAs in peripheral blood samples from four MDD patients and four healthy controls. A total of 44,787 m^6^A-modified mRNAs and 8,078 m^6^A-modified lncRNAs were detected (Figures 1A,B). Differentially expressed and differentially m^6^A-methylated RNAs were identified using a threshold of fold change ≥1.5 and p < 0.05.

To identify transcripts with significant differential expression and m^6^A methylation changes, we applied a threshold of fold change ≥1.5 and p < 0.05. Compared with healthy controls, the MDD group exhibited 171 significantly upregulated and 179 downregulated mRNAs (Figure 1C), as well as 71 significantly upregulated and 39 downregulated lncRNAs (Figure 1D). Regarding m^6^A methylation status, 180 mRNAs and 58 lncRNAs were hypermethylated, whereas 333 mRNAs and 61 lncRNAs were hypomethylated in the MDD group relative to controls (Figures 1E,F).

To further explore the directional relationship between m^6^A methylation changes and mRNA expression changes, we constructed a quadrant plot (Figure 1G) using a more permissive filtering strategy: transcripts with fold change ≥1.5 for m^6^A methylation (without p-value requirement) and fold change ≥1.5 for mRNA expression (without p-value requirement) were included. This permissive approach prioritizes sensitivity for pattern discovery and complements the stricter statistical filtering used in the volcano plot. Under these criteria, 557 mRNAs were classified as hypermethylated and upregulated (Hyper/Up), and 1,067 as hypomethylated and downregulated (Hypo/Down).

Among the 350 differentially expressed mRNAs, several were notably associated with depression susceptibility, including *GRM4*, *CAMKK2*, *SCN1A*, and *ATRN*. The ten most significantly upregulated and downregulated mRNAs are listed in Table 2.

**TABLE 2:** Significantly upregulated and downregulate mRNAs.

GeneSymbol	Transcript_ID	FC	P	FDR	Regulation
NIPAL3	ENST00000339255	3.486	0.048	0.933	Up
TMEM144	ENST00000514558	3.402	0.010	0.933	Up
NXNL2	ENST00000375855	2.811	0.036	0.933	Up
SCN1A	NM_001353950	2.795	0.025	0.933	Up
AFF3	ENST00000317233	2.725	0.043	0.933	Up
PSG7	ENST00000446844	2.653	0.049	0.933	Up
RBP1	ENST00000483943	2.649	0.010	0.933	Up
CATG00000053945.1	ENCT00000263490	2.629	0.033	0.933	Up
DUXA	ENST00000554048	2.608	0.037	0.933	Up
DPH6	ENST00000256538	2.576	0.020	0.933	Up
RDH11	ENST00000381346	0.283	0.023	0.933	Down
CFAP206	ENST00000369562	0.290	0.008	0.933	Down
CPLX2	ENST00000393745	0.306	0.012	0.933	Down
FOXA2	ENST00000377115	0.330	0.029	0.933	Down
STK26	ENST00000496850	0.337	0.006	0.933	Down
CAMK2N1	ENST00000375078	0.343	0.039	0.933	Down
VRK3	ENST00000599538	0.359	0.011	0.933	Down
CAB39	ENST00000410084	0.382	0.019	0.933	Down
GABRA2	ENST00000356504	0.385	0.009	0.933	Down
RBP2	ENST00000232217	0.385	0.037	0.933	Down

n = 4 per group (MDD, and healthy controls); FC, fold change; FDR, false discovery rate (Benjamini–Hochberg adjusted); Up, upregulated; Down, downregulated; Statistical criteria: Differential expression analysis was performed using the Limma package with empirical Bayes moderation. Transcripts with fold change ≥1.5 and nominal p < 0.05 were considered differentially expressed.

Our study identified 110 differentially expressed lncRNAs, such as *ELAVL4*, *PAX8-AS1*, and *TTC28-AS1*. Table 3 presents the top 20 lncRNAs that showed the most significant changes.

**TABLE 3:** Significantly upregulated and downregulate lncRNAs.

GeneSymbol	Transcript_ID	FC	P	FDR	Regulation
RP11-244H18.1	ENST00000621705	3.632	0.029	0.813	Up
ELAVL4	NR_136725	3.246	0.033	0.813	Up
AC112229.7	NR_027244	3.059	0.028	0.813	Up
RP11-187C18.2	NR_148348	3.006	0.014	0.813	Up
CTC-523E23.6	ENST00000595783	2.771	0.038	0.813	Up
ZNF385B	NR_148059	2.764	0.048	0.813	Up
CAV2	ENST00000477018	2.743	0.043	0.813	Up
RP11-269F20.1	ENST00000439849	2.621	0.036	0.813	Up
RP11-21F16.1	ENST00000591381	2.542	0.017	0.813	Up
RP11-551L14.5	ENST00000500527	2.444	0.023	0.813	Up
SLC35E2	ENST00000246421	0.362	0.046	0.813	Down
RP11-485G7.6	ENST00000574681	0.378	0.018	0.813	Down
PAX8-AS1	ENST00000445745	0.416	0.018	0.813	Down
RP11-490D19.6	ENST00000425734	0.419	0.006	0.813	Down
AP000288.2	ENST00000453716	0.439	0.017	0.813	Down
RARS2	NR_146741	0.447	0.042	0.813	Down
RP4-792G4.2	ENST00000449386	0.454	0.029	0.813	Down
TTC28-AS1	ENST00000426594	0.456	0.000	0.813	Down
RP11-18H21.1	ENST00000509332	0.472	0.036	0.813	Down
TYSND1	NR_073582	0.479	0.047	0.813	Down

n = 4 per group (MDD, and healthy controls); FC, fold change; FDR, false discovery rate (Benjamini–Hochberg adjusted); Up, upregulated; Down, downregulated; Statistical criteria: Differential expression analysis was performed using the Limma package with empirical Bayes moderation. Transcripts with fold change ≥1.5 and nominal p < 0.05 were considered differentially expressed.

Furthermore, 513 mRNAs exhibited alterations in m^6^A methylation modifications, including *SCN1A*, *FBXL13*, *Cplx2*, *GRM4*, and *CAMKK2*. Table 4 summarizes the ten mRNAs with the most significant hypermethylation and hypomethylation.

**TABLE 4:** mRNAs with m^6^A significant hypermethylation and hypomethylation.

GeneSymbol	Transcript_ID	FC	P	FDR	Regulation
SCN1A	NM_001353950	5.250	0.020	0.956	Hyper
RBP1	ENST00000483943	4.606	0.024	0.956	Hyper
NIPAL3	ENST00000339255	4.328	0.037	0.956	Hyper
TMEM144	ENST00000514558	4.133	0.009	0.956	Hyper
CATG00000117912.1	ENCT00000054505	4.113	0.037	0.956	Hyper
PSG7	ENST00000446844	3.749	0.040	0.956	Hyper
FBXL13	ENST00000436908	3.565	0.017	0.956	Hyper
DUXA	ENST00000554048	3.538	0.024	0.956	Hyper
OR4Q3	ENST00000642117	3.330	0.043	0.956	Hyper
ATRN	ENST00000446916	3.327	0.026	0.956	Hyper
FOXA2	ENST00000377115	0.164	0.030	0.956	Hypo
RDH11	ENST00000381346	0.187	0.008	0.956	Hypo
CPLX2	ENST00000393745	0.190	0.021	0.956	Hypo
ANKZF1	ENST00000410034	0.195	0.021	0.956	Hypo
USP14	ENST00000582707	0.196	0.002	0.956	Hypo
CAB39	ENST00000410084	0.205	0.050	0.956	Hypo
C18orf21	ENST00000610527	0.228	0.039	0.956	Hypo
GOLGA7	ENST00000520817	0.230	0.004	0.956	Hypo
STRA13	ENST00000392359	0.232	0.012	0.956	Hypo
C9orf43	ENST00000374165	0.235	0.019	0.956	Hypo

n = 4 per group (MDD, and healthy controls); FC, fold change; FDR, false discovery rate (Benjamini–Hochberg adjusted); Hyper, hypermethylated (higher m^6^A level in MDD); Hypo, hypomethylated (lower m^6^A level in MDD); Statistical criteria: Differential m^6^A methylation was performed using the Limma package with empirical Bayes moderation. Transcripts with fold change ≥1.5 and nominal P < 0.05 were considered differentially methylated.

Additionally, 119 lncRNAs were modified by m^6^A methylation, including *ELAVL4* and *SLC25A46*. Table 5 details the ten lncRNAs showing the most significant hypermethylation and hypomethylation.

**TABLE 5:** m^6^A lncRNAs with significant hypermethylation and hypomethylation.

GeneSymbol	Transcript_ID	FC	P	FDR	Regulation
RP11-244H18.1	NR_047603	5.300	0.030	0.911	Hyper
AC112229.7	NR_104388	4.238	0.031	0.911	Hyper
AC112229.7	NR_148503	4.238	0.031	0.911	Hyper
ZNF385B	ENST00000434868	4.197	0.023	0.911	Hyper
RP11-442J17.2	ENST00000553312	4.058	0.033	0.911	Hyper
RP11-187C18.2	ENST00000423715	3.852	0.019	0.911	Hyper
ELAVL4	ENST00000449316	3.354	0.039	0.911	Hyper
CTC-523E23.6	NR_136555	3.204	0.048	0.911	Hyper
RP4-760C5.5	NR_122111	3.016	0.016	0.911	Hyper
RP11-290F5.1	ENST00000432386	2.967	0.047	0.911	Hyper
TTC28-AS1	ENST00000426594	0.187	0.005	0.911	Hypo
PAX8-AS1	ENST00000445745	0.213	0.025	0.911	Hypo
C11orf36	ENST00000434798	0.233	0.004	0.911	Hypo
DSCAM	NR_073202	0.249	0.016	0.911	Hypo
RARS2	NR_146741	0.259	0.035	0.911	Hypo
GPR78	ENST00000504255	0.276	0.004	0.911	Hypo
TYSND1	NR_073582	0.279	0.039	0.911	Hypo
RP11-48F14.1	ENST00000441169	0.298	0.004	0.911	Hypo
CTC-459F4.3	NR_110690	0.346	0.018	0.911	Hypo
LOC100288069	NR_033908	0.347	0.032	0.911	Hypo

n = 4 per group (MDD, and healthy controls); FC, fold change; FDR, false discovery rate (Benjamini–Hochberg adjusted); Hyper, hypermethylated (higher m^6^A level in MDD); Hypo, hypomethylated (lower m^6^A level in MDD); Statistical criteria: Differential m^6^A methylation was performed using the Limma package with empirical Bayes moderation. Transcripts with fold change ≥1.5 and nominal p < 0.05 were considered differentially methylated.

In addition, to further quantify the relationship between m^6^A methylation and transcript abundance, we performed Pearson correlation analysis between methylation fold change (log_2_FC of m^6^A methylation) and expression fold change (log_2_FC of transcript expression) for all overlapping transcripts. Strong positive correlations were observed for both mRNAs and lncRNAs (r = 0.831 and 0.853 respectively, both p < 0.001), indicating that changes in m^6^A methylation levels are closely associated with corresponding changes in transcript abundance.

### 3.2 Functional enrichment analysis of differentially expressed mRNAs

**FIGURE 2:**
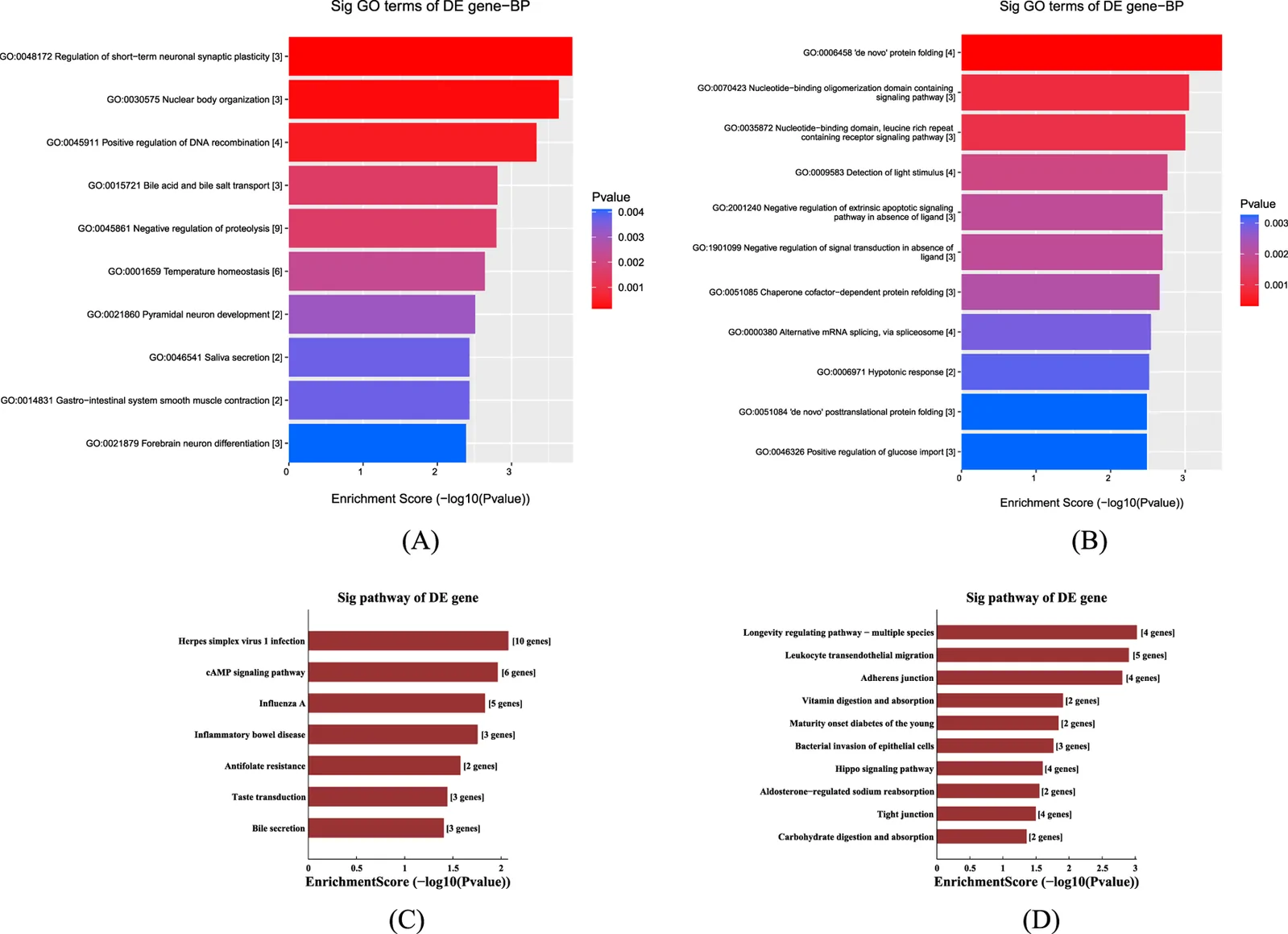
GO and KEGG analyses of differentially expressed mRNAs. **(A)** The top ten enriched items obtained from GO analysis of upregulated mRNAs with differential expression. **(B)** The top ten enriched items obtained from GO analysis of downregulate mRNAs with differential expression. **(C)** The first ten enriched pathways identified in KEGG analysis of upregulated mRNAs with differential expression. **(D)** The first ten enriched pathways identified in KEGG analysis of downregulate mRNAs with differential expression. Note: n = 4 per group. Gene ontology analysis associates the differentially m6A-methylated mRNAs enriched in certain gene ontological functions and GO terms (http://www.geneontology.org). The statistical significance of the enrichment is calculated by Fisher Exact test p-value and also -log10(p) transformed as the enrichment score. The lower the p-value or the higher the enrichment, the better the statistical significance of differentially m6A-methylated RNAs in that function. Pathway analysis associates the differentially m6A-methylated mRNAs enriched in certain biological pathways. The statistical significance of the enrichment is calculated by Fisher Exact test p-value and also -log10(p) transformed as the enrichment score. The lower the p-value or the higher the enrichment, the better the statistical significance of differentially m6A-methylated mRNAs in that pathway.

As illustrated in Figure 2, Gene Ontology (GO) analysis revealed distinct functional enrichment patterns between upregulated and downregulated mRNAs. Upregulated mRNAs were primarily associated with biological processes, including regulation of short-term neuronal synaptic plasticity, organization of nuclear bodies, and positive regulation of DNA recombination (Figure 2A). In contrast, downregulated mRNAs were mainly enriched in *de novo* protein folding, nucleotide-binding oligomerization domain (NOD)-containing signaling pathways, and nucleotide-binding domain, leucine-rich repeat-containing receptor signaling pathways (Figure 2B).

KEGG pathway analysis further demonstrated distinct enrichment profiles. Upregulated mRNAs were predominantly enriched in pathways related to herpes simplex virus 1 infection, the cAMP signaling pathway, and influenza A (Figure 2C). Conversely, downregulated mRNAs were mainly associated with longevity regulation across multiple species, leukocyte transendothelial migration, and adherens junctions (Figure 2D).

### 3.3 Functional analyses of differentially m^6^A modified mRNAs

**FIGURE 3:**
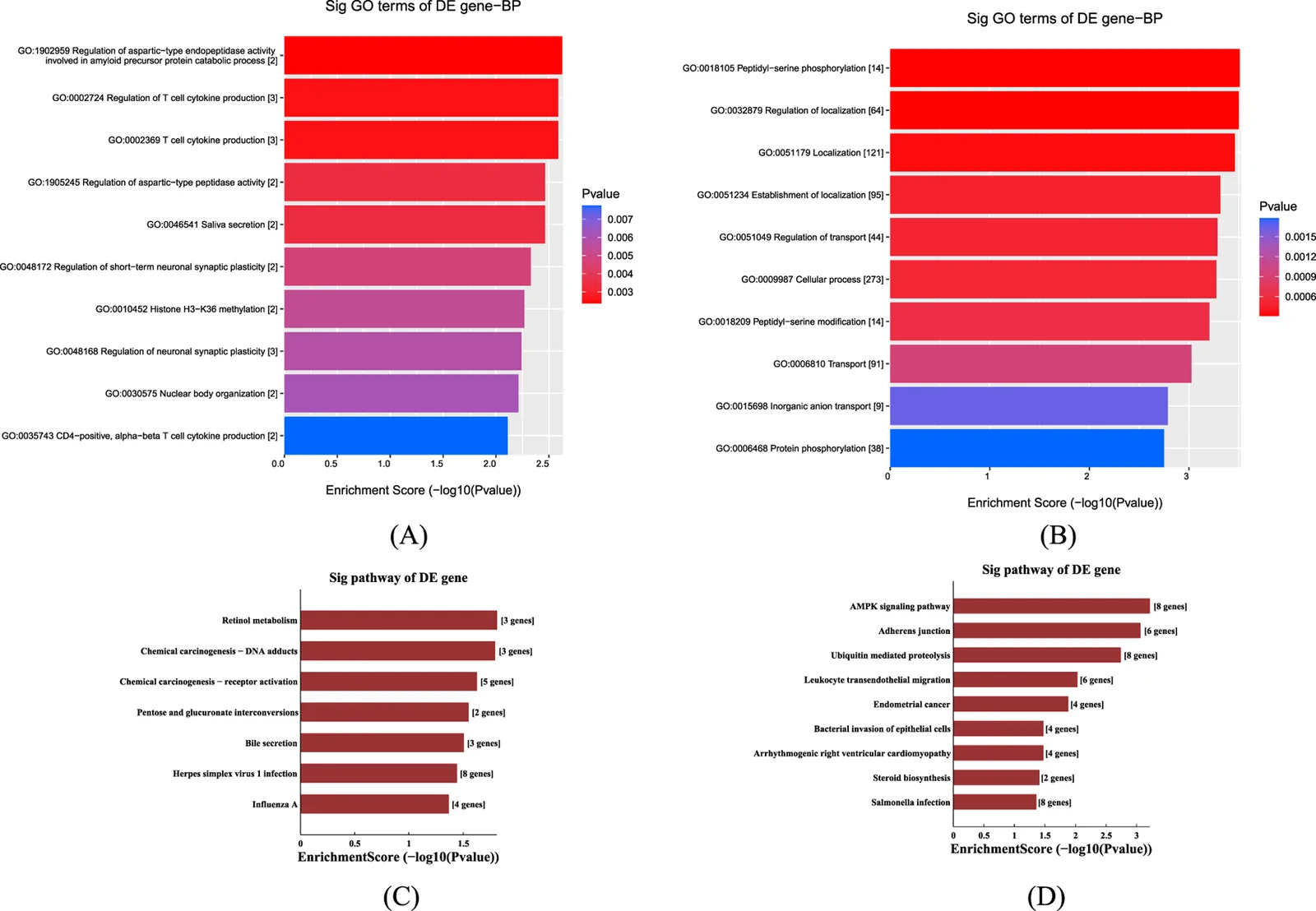
GO and KEGG analyses of differentially methylated mRNAs **(A)** The top ten enriched items obtained from GO analysis of differentially hypermethylated mRNAs. **(B)** Top ten enriched items obtained from GO analysis of differentially hypomethylated mRNAs. **(C)** The first ten enriched pathways identified in KEGG analysis of differentially hypermethylated mRNAs. **(D)** The top ten enriched pathways identified in KEGG analysis of differentially hypomethylated mRNAs. Note: n = 4 per group. Gene ontology analysis associates the differentially m6A-methylated mRNAs enriched in certain gene ontological functions and GO terms (http://www.geneontology.org). The statistical significance of the enrichment is calculated by Fisher Exact test p-value and also -log10(p) transformed as the enrichment score. The lower the p-value or the higher the enrichment, the better the statistical significance of differentially m6A-methylated RNAs in that function. Pathway analysis associates the differentially m6A-methylated mRNAs enriched in certain biological pathways. The statistical significance of the enrichment is calculated by Fisher Exact test p-value and also -log10(p) transformed as the enrichment score. The lower the p-value or the higher the enrichment, the better the statistical significance of differentially m6A-methylated mRNAs in that pathway.

As shown in Figure 3, Gene Ontology (GO) analysis revealed distinct functional enrichment patterns between hypermethylated and hypomethylated mRNAs. Hypermethylated mRNAs were predominantly enriched in biological processes related to aspartate-type endopeptidase activity involved in amyloid precursor protein catabolism, T-cell cytokine production, and regulation of T-cell cytokine production (Figure 3A). In contrast, hypomethylated mRNAs were primarily associated with peptidyl-serine phosphorylation, localization regulation, and transport mechanisms (Figure 3B).

KEGG pathway analysis further demonstrated distinct enrichment profiles (Figure 3C). Hypermethylated mRNAs were primarily involved in retinol metabolism, chemo-attenuation DNA adduct formation, and neuroactive ligand-receptor interactions. Conversely, hypomethylated mRNAs were mainly linked to the AMPK signaling pathway, adherens junctions, and ubiquitin-mediated proteolysis (Figure 3D).

### 3.4 Validation of differentially m^6^A-Methylated RNAs by MeRIP-qPCR

Candidate transcripts for MeRIP-qPCR validation were selected from the microarray discovery results using a three-step prioritization strategy. First, transcripts showing both significant differential m^6^A methylation (fold change ≥1.5, p < 0.05) and differential expression (fold change ≥1.5, p < 0.05) were retained. Second, among these, we prioritized transcripts mapping to the two core KEGG pathways identified by our enrichment analysis, neuroactive ligand-receptor interactions and the AMPK signaling pathway. Third, we selected two lncRNAs (*PAX8-AS1* and *TTC28-AS1*) based on their differential methylation and expression patterns. This yielded four candidates: two mRNAs (*GRM4* and *CAMKK2*) and two lncRNAs (*PAX8-AS1* and *TTC28-AS1*). Validation was performed in an independent cohort comprising 15 MDD patients and 15 healthy controls.

**FIGURE 4:**
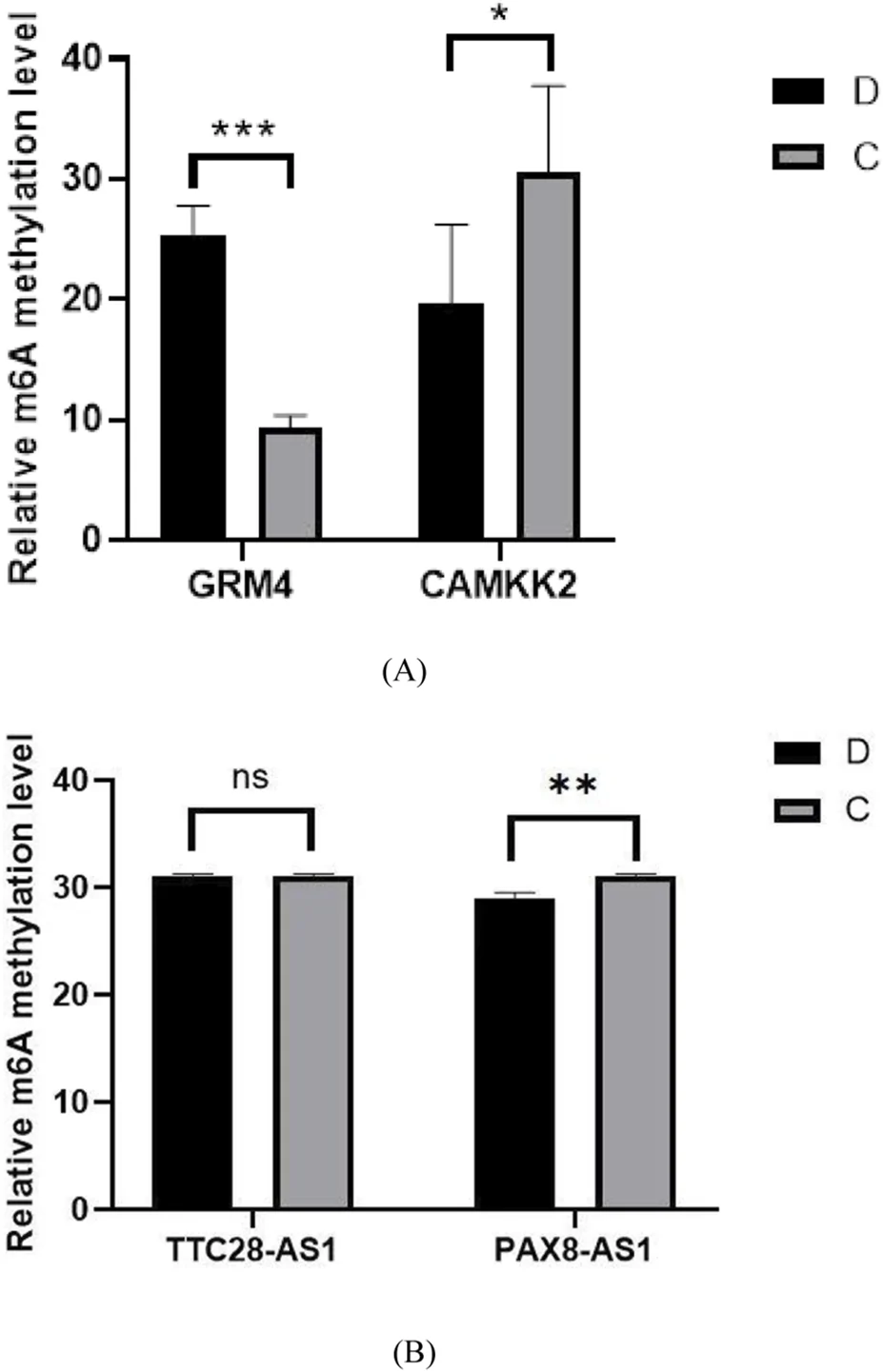
Validations of the m^6^A methylation level and expression level of the mRNAs and lncRNAs by MeRIP-qPCR. **(A)** Relative m^6^A methylation level of mRNA (*GRM4* and *CAMKK2*) in patients with MDD by MeRIP-qPCR. **(B)** Relative m^6^A methylation level of lncRNAs (*TTC28-AS1* and *PAX8-AS1*) in patients with MDD by MeRIP-qPCR. Note: n = 15 per group (MDD and healthy controls); *GRM4*, glutamate metabotropic receptor 4; *CAMKK2*, calcium/calmodulin-dependent protein kinase kinase 2; *PAX8-AS1*, PAX8 antisense RNA 1; *TTC28-AS1*, *TTC28* antisense RNA1; Statistical significance was evaluated using Student’s t-test; *p < 0.05; **p < 0.01; ***p < 0.001; ns, p > 0.05.

As shown in Figure 4A, the m^6^A methylation level of *GRM4* was significantly higher in MDD patients than in healthy controls (p < 0.001). In contrast, *CAMKK2* exhibited a significantly lower methylation level in the MDD group (p = 0.012). Regarding lncRNAs, the methylation level of *PAX8-AS1* was significantly lower in MDD patients compared with controls (p = 0.006), whereas no significant difference was observed for *TTC28-AS1* between the two groups (Figure 4B). These validation results were largely consistent with the microarray data, further supporting the reliability of our findings.

## 4 Discussion

In this study, we systematically characterized m^6^A epitranscriptomic landscape of mRNAs and lncRNAs in peripheral blood from first-episode, treatment-naïve patients with major depressive disorder (MDD) through integrated methylome and transcriptome profiling. Our main findings are as follows: (1) widespread m^6^A methylation alterations were observed in MDD, involving 513 mRNAs and 119 lncRNAs; (2) differentially methylated RNAs were significantly enriched in neuroactive ligand-receptor interaction and AMPK signaling pathways; (3) m^6^A methylation changes were associated with altered gene expression; and (4) *GRM4*, *CAMKK2*, and *PAX8-AS1* were validated as differentially m^6^A-methylated RNAs in an independent cohort. Collectively, these findings suggest that peripheral m^6^A methylation signatures are associated with MDD and may represent promising candidate biomarkers for further evaluation, while the implicated pathways provide hypothesis-generating avenues for future mechanistic and therapeutic investigation.

### 4.1 Association of m^6^A modification with the immune-inflammation hypothesis in MDD

GO enrichment analysis revealed that differentially m^6^A-methylated mRNAs were significantly involved in T-cell cytokine production, regulation of short-term neuronal synaptic plasticity, and modulation of synaptic plasticity. These findings provide epitranscriptomic evidence supporting the immune-inflammation hypothesis of MDD. Previous studies have demonstrated altered T cell and B cell subsets in peripheral blood of MDD patients (Beurel et al., 2022), and adaptive immune responses are known to influence neural plasticity, thereby contributing to the pathogenesis of depression (Ghosh et al., 2021). Our study extends these observations by implicating m^6^A modification of immune-related genes as a potential upstream regulatory mechanism underlying immune dysfunction in MDD. Although the role of m^6^A in T cell activation and cytokine production has been documented in other diseases, our study is among the first to link m^6^A-modified immune-related transcripts to MDD, offering a novel perspective on the neuroimmune crosstalk in this disorder.

### 4.2 m^6^A modification participates in MDD pathogenesis via AMPK and neuroactive ligand-receptor pathways

KEGG pathway analysis indicated that differentially m^6^A-methylated mRNAs were predominantly enriched in neuroactive ligand-receptor interaction and AMPK signaling pathways, both of which are intimately associated with MDD pathogenesis. We further validated key molecules representing these pathways: *GRM4*, a core component of the neuroactive ligand-receptor pathway, exhibited hypermethylation in the MDD group. *GRM4* is predominantly localized at presynaptic terminals, where it negatively regulates glutamate release and maintains central nervous system homeostasis (Stansley and Conn, 2019). Previous studies have reported upregulated *GRM4* expression in MDD patients (Chandley et al., 2014; Lopez et al., 2014) and its involvement in MDD through miRNAs such as miR-1202 (Lopez et al., 2014) and miR-335 (Li et al., 2015). Our findings reveal an association between aberrant m^6^A methylation and altered expression of *GRM4* in peripheral blood from MDD patients, suggesting a potential epigenetic regulatory link that requires validation in brain-relevant systems. *CAMKK2*, an upstream activator of the AMPK signaling pathway, showed hypomethylation in the MDD group. *CAMKK2* activates AMPK through phosphorylation, which in turn regulates downstream effectors such as *SIRT1* and *PGC-1α*. Activation of the AMPK/SIRT1 pathway has been shown to reduce pro-inflammatory cytokines and oxidative stress, exerting antidepressant effects (Liao et al., 2020). Similarly, the AMPK/PGC-1α/FNDC5/BDNF pathway has been implicated in antidepressant mechanisms (Hu et al., 2022). Although *CAMKK2* has received limited attention in MDD research, recent evidence suggests its potential as a diagnostic marker and its association with immune infiltration in MDD (Ning et al., 2022). Our study provides preliminary evidence of an association between m^6^A modification of *CAMKK2* in peripheral blood and MDD, though the nature of this relationship requires further investigation. Notably, the two validated mRNAs correspond to two core pathways (neuroactive ligand-receptor interaction and AMPK signaling), and the directionality of their m^6^A alterations (hypermethylation of *GRM4* and hypomethylation of *CAMKK2*) aligns with their reported expression patterns in MDD, reinforcing the biological coherence of our findings.

### 4.3 Potential role of m^6^A-Modified lncRNAs in MDD

This study represents the first systematic identification of differentially m^6^A-methylated lncRNAs in MDD, yielding 119 candidates, among which *PAX8-AS1* and *ELAVL4* were highlighted and partially validated. *PAX8-AS1* was validated to be hypomethylated in MDD blood. Previous studies have implicated *PAX8-AS1* as a key regulator of mRNA expression in schizophrenia (Sabaie et al., 2021) and as a functional methylation site in Parkinson’s disease (Henderson et al., 2021). Our findings extend its relevance to MDD, suggesting a broader role for this lncRNA in neuropsychiatric disorders. *ELAVL4*, the protein product of which is an RNA-binding protein that stabilizes mRNAs by binding to their 3′untranslated regions (Fabbri et al., 2015; Li et al., 2024), was found to be hypermethylated in MDD. *ELAVL4* polymorphisms have been associated with synaptic plasticity-related phenotypes (Fabbri et al., 2015), and neuron-specific ELAV-like proteins have been shown to alleviate anhedonia and behavioral despair in a mouse model of chronic mild stress (Sanna et al., 2018). The hypermethylation of *ELAVL4* observed in our study raises the possibility of altered RNA-processing function, which might hypothetically affect the expression of synaptic plasticity-related genes, though this remains to be tested in functional studies.

### 4.4 Explanation regarding the unvalidated molecule

One molecule, *TTC28-AS1*, did not reach statistical significance in MeRIP-qPCR validation. Several factors may account for this discrepancy. First, differences in detection principles between microarray-based m^6^A profiling (enrichment-based relative quantification) and MeRIP-qPCR (site-specific validation) may contribute to inconsistent results, as the two methods may capture distinct m^6^A-modified regions. Second, the relatively small validation sample size (n = 15 per group) may have limited statistical power to detect true differences, particularly given potential inter-individual heterogeneity. Third, m^6^A modification levels of *TTC28-AS1* may exhibit subgroup-specific patterns in MDD patients, warranting further investigation in larger cohorts stratified by clinical characteristics. Importantly, the three successfully validated molecules (*GRM4*, *CAMKK2*, and *PAX8-AS1*) show convergent enrichment in pathways previously implicated in MDD, including neuroactive ligand-receptor signaling, AMPK signaling, and RNA processing. This pattern provides preliminary support for the biological relevance of our findings, although the functional significance of these associations remains to be established.

Recently, Baldrighi et al. conducted a large-scale meta-analysis of DNA methylation in MDD, identifying hypomethylation near the *SHF* gene and 51 rare epivariations associated with the disorder (Baldrighi et al., 2024). Notably, no significant global DNA methylation differences were observed between MDD cases and controls, suggesting that the epigenetic alterations in MDD may be locus-specific rather than genome-wide. In contrast, our study revealed widespreadm^6^Amethylation changes affecting 1,475 mRNAs and 489 lncRNAs, with the AMPK signaling pathway and neuroactive ligand-receptor interactions being predominantly enriched. These findings suggest that while DNA methylation changes in MDD may be relatively focal, RNA methylation alterations appear more pervasive, potentially reflecting the dynamic nature of post-transcriptional regulation in response to environmental and stress-related stimuli. Together, these complementary epigenetic layers, DNA methylation at the genome level andm^6^Amodification at the transcriptome level, may converge on key pathways involved in synaptic plasticity and neuroimmune function.

To further explore the regulatory relationship between m^6^A methylation and transcript abundance, we performed correlation analyses across all overlapping transcripts. The strong positive correlations observed for both mRNAs and lncRNAs suggest a close association between methylation changes and expression changes. This finding provides quantitative support for the biological coherence of our m6A methylation signatures and reinforces the potential regulatory role of m6A modification in MDD. Nevertheless, given the cross-sectional and correlational nature of these analyses, causation cannot be inferred, and functional studies are required to establish direct regulatory mechanisms.

## 5 Limitations

Several limitations should be acknowledged. First, the sample size for the discovery microarray analysis was small (n = 4 per group), which limited statistical power and increased the likelihood of false-positive findings—a concern reflected in the high FDR values (>0.05) observed for most nominally significant transcripts. While the reliance on nominal P-values was justified for exploratory hypothesis generation and we employed empirical Bayes moderation to stabilize variance estimates, this approach inherently increases the risk of false positives. To mitigate this concern, we applied a stringent fold-change threshold (≥1.5) and validated selected candidates in an independent cohort (n = 15 per group). The successful validation of three out of four candidate transcripts (*GRM4*, *CAMKK2*, and *PAX8-AS1*) supports the robustness of our main findings. It should be emphasized that our study is exploratory and cross-sectional, and the observed associations are correlational rather than causal. While the identified candidates show promise, their utility as diagnostic biomarkers requires prospective validation with ROC-based performance analyses, and their therapeutic potential necessitates functional investigation in experimental models. Nevertheless, given the exploratory nature of the discovery phase, cautious interpretation is warranted, and future studies with larger discovery cohorts are essential to confirm the observed m^6^A methylation patterns. Therefore, these findings should be considered hypothesis-generating and provide a foundation for future studies rather than immediate clinical translation.

The second limitation of this study is the use of peripheral blood as the biological source. While peripheral blood offers practical advantages for biomarker discovery including non-invasive collection and clinical feasibility, it is well established that m^6^A methylation exhibits strong tissue specificity, and peripheral blood may not fully recapitulate m^6^A modification patterns in the brain. Therefore, the m^6^A methylation signatures identified in this study should be interpreted primarily as peripheral markers associated with MDD, rather than as direct evidence of brain-specific mechanisms. Indeed, the pathways we identified (e.g., neuroactive ligand-receptor interactions and AMPK signaling) may reflect systemic or immune-related processes rather than neuron-intrinsic changes. Future studies using brain tissue or cerebrospinal fluid are needed to determine whether similar m^6^A patterns are present in the central nervous system and to explore the tissue-specific dynamics of m^6^A modification in MDD.

Third, this study primarily describes m^6^A methylation differences and their association with gene expression, without establishing causality or directly demonstrating functional regulation of target RNAs by m^6^A modification. Fourth, mechanistic validation at the cellular or animal level is lacking, and the roles of m^6^A writers, erasers, and readers in mediating the observed methylation changes remain to be explored. Fourth, occupational and other socioeconomic variables, such as income, employment status, and shift work, were not collected in the current study. Although all participants were recruited from the same geographic region during the same time period, minimizing variability due to regional and seasonal factors, we acknowledge that occupational factors could theoretically influence RNA methylation through stress-related pathways. Future studies should incorporate detailed occupational and socioeconomic assessments to more comprehensively address potential confounders.

## 6 Conclusions and perspectives

In summary, this exploratory pilot study provides the first comprehensive characterization of the m^6^A epitranscriptomic landscape of mRNAs and lncRNAs in peripheral blood from first-episode, treatment-naïve MDD patients. Although the discovery sample size was small, the validation of key targets, including *GRM4*, *CAMKK2* and *PAX8-AS1*, in an independent cohort provides preliminary support for their relevance. Our findings suggest that m^6^A modification may contribute to MDD pathogenesis, potentially through the AMPK signaling pathway and neuroactive ligand-receptor interactions. *GRM4* and *PAX8-AS1* emerge as promising candidate transcripts meriting further validation in larger, well-powered cohorts, while the AMPK and neuroactive ligand-receptor pathways represent intriguing avenues for future mechanistic and therapeutic exploration, though causal relationships remain to be established. These results should be considered hypothesis-generating and require replication in larger cohorts. Future studies with functional characterization of m^6^A regulatory machinery (e.g., *METTL3*, *FTO*, *YTHDF* proteins) and mechanistic validation in relevant model systems are warranted further to elucidate the causal role of m^6^A methylation in MDD.

## Data Availability

The original contributions presented in the study are publicly available. This data can be found at Figshare with the doi 10.6084/m9.figshare.33442735, at https://doi.org/10.6084/m9.figshare.33442735.
